# Dual stimuli-responsive polyphosphazene-based molecular gates for controlled drug delivery in lung cancer cells†

**DOI:** 10.1039/d0ra03210g

**Published:** 2020-07-21

**Authors:** Yolanda Salinas, Michael Kneidinger, Cristina Fornaguera, Salvador Borrós, Oliver Brüggemann, Ian Teasdale

**Affiliations:** Institute of Polymer Chemistry (ICP), Johannes Kepler University Linz (JKU) Altenberger Strasse 69 4040 Linz Austria yolanda.salinas@jku.at; Linz Institute of Technology (LIT), Johannes Kepler University Linz (JKU) Altenberger Strasse 69 4040 Linz Austria; Grup d'Enginyeria de Materials (GEMAT), Institut Químic de Sarrià (IQS), Universitat Ramon Llull (URL) Via Augusta 390 Barcelona 08017 Spain

## Abstract

A switchable silane derived stimuli-responsive bottle-brush polyphosphazene (PPz) was prepared and attached to the surface of mesoporous silica nanoparticles (MSNs). The hybrid polymer with PEG-like Jeffamine® M-2005 side-arms undergo conformational changes in response to both pH and temperature due to its amphiphilic substituents and protonatable main-chain, hence were investigated as a gatekeeper. Safranin O as control fluorophore or the anticancer drug camptothecin (CPT) were encapsulated in the PPz-coated MSNs. At temperatures below the lower critical solution temperature (LCST), the swollen conformation of PPz efficiently blocked the cargo within the pores. However, above the LCST, the PPz collapsed, allowing release of the payload. Additionally, protonation of the polymer backbone at lower pH values was observed to enhance opening of the pores from the surface of the MSNs and therefore the release of the dye. *In vitro* studies demonstrated the ability of these nanoparticles loaded with the drug camptothecin to be endocytosed in both models of tumor (A549) and healthy epithelial (BEAS-2B) lung cells. Their accumulation and the release of the chemotherapeutic drug, co-localized within lysosomes, was faster and higher for tumor than for healthy cells, further, the biocompatibility of PPz-gated nanosystem without drug was demonstrated. Tailored dual responsive polyphosphazenes thus represent novel and promising candidates in the construction of future gated mesoporous silica nanocarriers designs for lung cancer-directed treatment.

## Abstract 2

Bottle-brush polyphosphazenes as dual, thermosensitive and pH responsive gatekeepers for mesoporous silica nanoparticles, and their use in controlled drug release.

## Graphical abstract

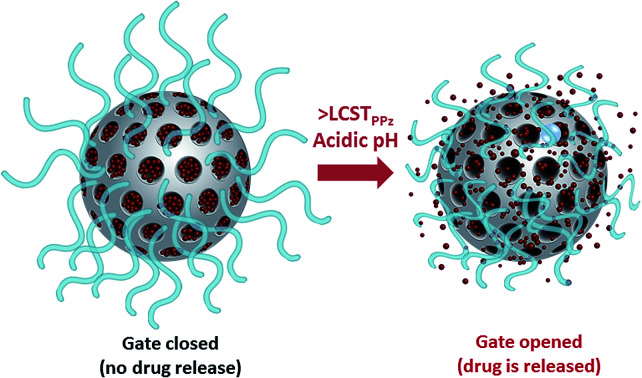

## 1. Introduction

Macromolecular materials can undergo physical changes in response to external stimuli, for example light, pH, temperature, oxidation and magnetic fields.^1,2^ Sometimes referred to as smart or stimuli-responsive polymers,^3^ such materials have wide ranging applications as chemo- and biosensors,^4^ chemo-mechanical actuators^5^ and controlled release applications.^6^ For the release of bioactives, phosphorus based polymers offer promise due to their capability to be fine-tuned, for their biodegradability and multivalency.^7^ Of these, polyphosphazenes^8^ are quite advanced in biomedical applications,^9,10^ with fluorinated derivatives having FDA approval as stent coatings^11^ and vaccine adjuvants in advanced clinical trials.^12,13^ Thermoresponsive polyphosphazenes have been extensively investigated, in particular for their use as thermosensitive injectable hydrogels, with, among others, delivery or siRNA for gene silencing, stem cell delivery^14^ and combinatorial cancer therapy.^15^ Due to a widely observed phenomenon for amphiphilic polymers,^16,17^ thermoresponsive polymers commonly possess a lower critical solution temperature (LCST) in aqueous solutions, above which the macromolecules collapse, followed often by agglomeration and/or gelation.^17^ On surfaces the macromolecules collapse,^18^ for example to control fuel access to micromotors^19^ or to modulate cell adhesion and detachment, facilitating fabrication of cell sheets.^20^ In this work, we use Jeffamine-functionalized bottle-brush polyphosphazenes which are reported to have LCSTs which can be tuned to biologically relevant temperatures.^21^ The multivalent nature allows for chemical grafting to surfaces while the amphiphilic character is used to adjust LCST.^22^ Furthermore, since the basic nitrogen atoms in the polyphosphazene backbone become protonated in acidic media, pH can also be a stimulus, influencing the polymer polarity, LCST and potentially conformation.^23^

The incorporation of this polymer to the surface of mesoporous silica nanoparticles (MSNs) will increase the nanocarriers stability, maximizing the “stealth” features of this novel system and reducing opsonization, all potentially prolonging the blood circulation of the material. The very interesting properties of MSNs, such as thermal stability, easy chemical functionalization or rigid framework which prevents premature degradation of the cargo, make them stand out as synthetic platforms in the design of hybrid materials.^24^ Among many applications, gated mesoporous silica nanoparticles have been promoted mainly in biomedicine^25,26^ because these systems are promising candidates for developing new, more efficient and safety improved therapies, setting a precedent in the area of drug delivery.^27^ The external functionalization of the pores with stimuli-responsive hydrophilic polymeric gate-keeper units by easy “click” post-grafting strategy^28^ permits the controlled release upon application of different stimuli while enhancing their stability in aqueous environments. Therefore, we designed and report herein hybrid silica-based nanomaterials containing molecular gates based on polyphosphazene polymer units sensitive to both temperature and pH to control more precisely the delivery of chemotherapeutics through a selective and reversible “close-opening gate mechanism”.

## 2. Results and discussion

### 2.1. pH and temperature responsive nanosystem design and gated mechanism

**Fig. 1:**
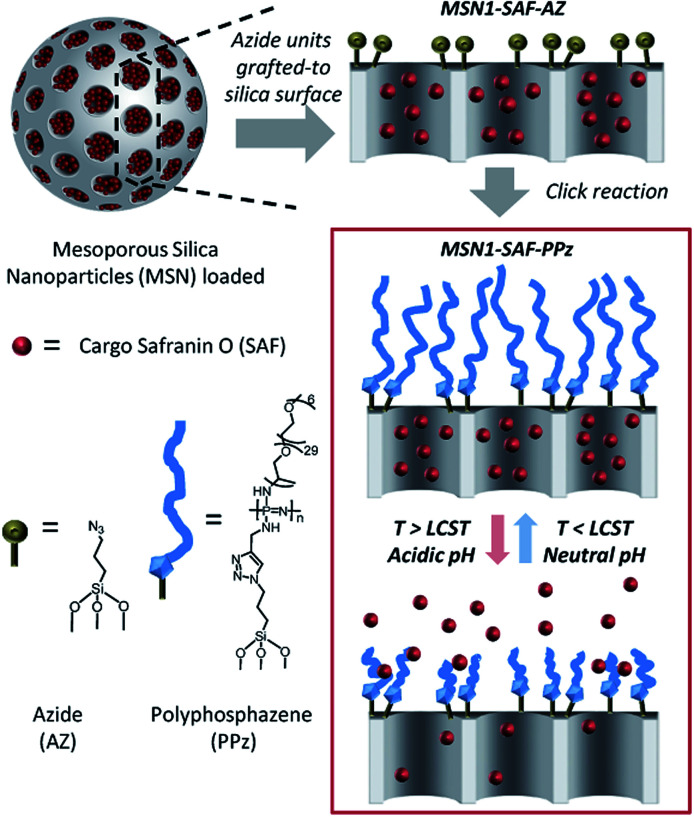
Schematic illustration of the polymer functionalization on the silica surface and the cargo controlled release mechanism of dual temperature and pH responsive gated polyphosphazene units (PPz). The material was initially uploaded with safranin O and grafted with azide moieties (MSN1-SAF-AZ) following the attachment of the polymer to the surface of silica mesopores *via* click chemistry (MSN1-SAF-PPz). The scheme of PPz shows the simplified structure of the statistically distributed Jeffamine® M-2005 moieties along the polymer backbone (*n* ∼ 50). Cargo release occurs by increasing the temperature above LCST of PPz due to collapsing of the polymer and opening the pores.

A schematic representation of the pH and thermoresponsive polyphosphazene-based molecular gate nanosystem for controlled drug delivery is shown in Fig. 1. Initially, mesoporous silica nanoparticles (MSNs) were synthesized to act as inorganic carrier following the well-known sol–gel process. For this, the silica precursor tetraethylorthosilicate (TEOS) forms around the cationic surfactant cetyltrimethyl ammonium bromide (CTAB) as pore-template agent under alkaline conditions carried out as reported in literature.^29^ The mesoporous nanoparticles were obtained by calcination at 550 °C (so called here nanoparticles MSN0).

The empty porous nanoparticles were then loaded with the dye safranin O as suitable cargo unit. The outer pores surface of the loaded MSNs was functionalized with 3-(azidopropyl)triethoxysilane groups (nanoparticles MSN1-SAF-AZ labelled according to the corresponding loaded cargo) to subsequently be added by a copper catalysed Huisgen cycloaddition reaction^28,30^ to previously synthetized polyphosphazenes containing Jeffamine® M-2005 and alkyne moieties (polymer called here as PPz). The resulting nanoparticles were isolated by centrifugation, washed with water and acetonitrile to remove any residual cargo, possible catalyst traces and ungrafted polymer moieties. A final drying step under vacuum yielded the nanomaterial MSN1-SAF-PPz.

### 2.2. Characterization of the gatekeeper polyphosphazene

**Fig. 2:**
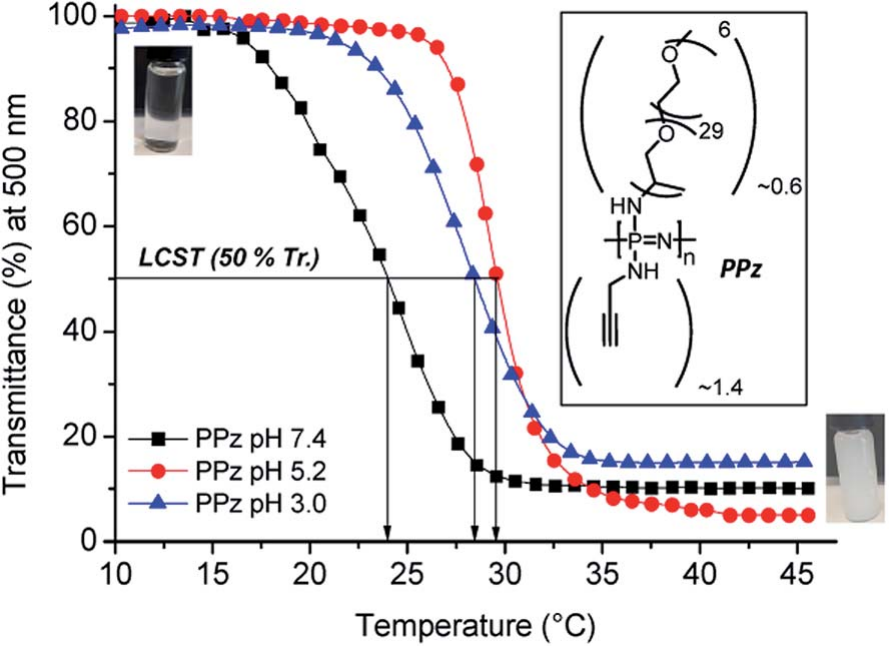
Lower critical solution temperature (LCST) of Jeffamine® M-2005 and Jeffamine containing polyphosphazene (PPz) (*n* ∼ 50) determined by transmittance measurements with increasing temperature at 500 nm for 1 mg mL^−1^ of the polymer in phosphate buffer solution at pH 7.4, 5.2 and acidic solution of pH 3.0, inset: chemical structure of polymer PPz and corresponding images of the polymer solution (1 mg mL^−1^) below (transparent solution) and above LCST (collapsed state and agglomerated polymer chains) at neutral pH.

A bottle-brush polyphosphazene was prepared containing two different substituents, hydrophilic PEO-PPO-NH_2_ oligomers (Jeffamine® M-2005) and 2-propynylamine in molar ratios 1 : 2, confirmed by ^1^H-NMR spectroscopy (see ESI†). Initially, a precursor poly(dichloro)phosphazene [NPCl_2_]_*n*_ with 50 repeating units (estimated by ^31^P NMR, see ESI†) was prepared *via* phosphine-mediated living polymerization of Cl_3_P=N–SiMe_3_, as reported in the literature.^31^ The chlorine atoms of this precursor were then substituted with 2-propynylamine and with Jeffamine® M-2005 yielding the final polyphosphazene (termed PPz, see structure in Fig. 2 inset).

The LCST of PPz was determined by UV-Vis spectroscopy at different pH and temperature conditions (Fig. 2). Initially, the inherent thermoresponsive of Jeffamine® M-2005 was measured at neutral and acidic pHs and a negligible increase of 1 °C, in the range of 21–22 °C, from PBS solutions at pH 7.4 to 5.2 (see Fig. SI-2, ESI†). The same procedure was repeated for PPz, up to pH 3.0. Interestingly, the PPz showed pH dependent response, with a shift towards higher temperatures, from 24 to 28–29 °C upon moving from neutral to acidic conditions (LCST determination at 50% transmittance, see Fig. 2). That was in agreement to the expected overall increase of the hydrophilicity of the polymer due to protonation of the N atoms from the PPz backbone at acidic pHs.^19^

### 2.3. Characterization of the hybrid nanosystems

**Fig. 3:**
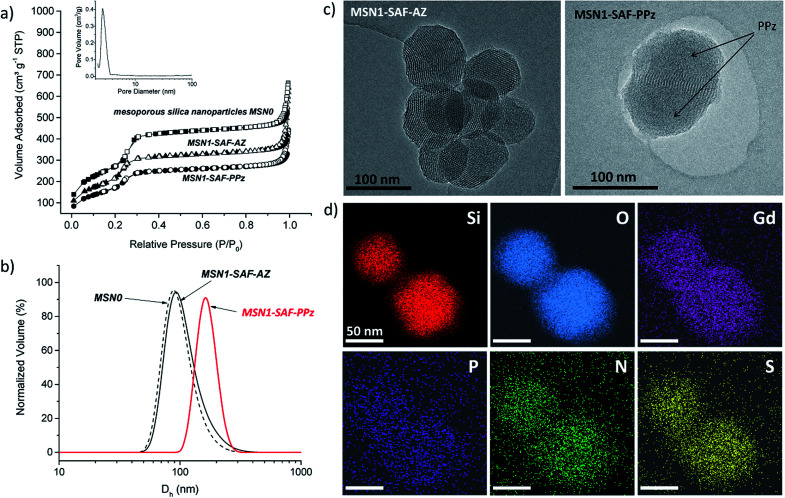
(a) Nitrogen adsorption–desorption isotherms of materials MSN0, MSN1-SAF-AZ and MSN1-SAF-PPz. Full symbols and empty symbols stand for adsorption and desorption respectively, inset: BJH pore size distribution of MSN0; (b) dynamic light scattering (DLS) measurements (hydrodynamic diameter, *D*_h_, *via* normalized volume) of MSN0, MSN1-SAF-AZ and MSN1-SAF-PPz (1 mg mL^−1^ aqueous suspension); (c) TEM images of the safranine-loaded nanoparticles MSN1-SAF-AZ and MSN1-SAF-PPz, where the polymer was located on the surrounding of the silica surface blocking the pores, marked by arrows in the right image. Scale bar: 100 nm; (d) TEM-EDX mapping images of nanoparticles MSN1-SAF-PPz stained with Uranyless (uranium-free staining contrast solution). Gd element was used as contrast agent, highly staining the organic parts of the system. Scale bar: 50 nm.

The morphology of the prepared hybrid mesoporous silica nanoparticles was initially determined by transmission electron microscopy (TEM). The TEM micrograph of the mesoporous silica nanoparticles containing the dye and functionalized with azide groups (MSN1-SAF-AZ) showed homogeneous spherical shape and external smooth surface, with sizes below 120 nm. The hexagonal and longitudinal arrangement of regular pores observed was in agreement with similar previously reported MCM-41 type nanoparticles porosity (typical repeated mesopores are shown as black and white stripes in Fig. 3c).^32^

In order to confirm this, their hydrodynamic diameter was estimated by dynamic light scattering in aqueous suspension. The data collected in Table 1 was in agreement with the previous microscope images. A negligible difference in hydrodynamic diameter from calcined MSN0 to intermediate nanomaterials MSN1-SAF-AZ was estimated where the nanoparticles kept their size distribution after dye loading and functionalization with azides (*D*_h_ between 108–110 nm). Besides, a higher increase in size of *ca.* 40 nm was detected for the polymer functionalized nanomaterial MSN1-SAF-PPz, observed also in the TEM micrograph (see Fig. 3c). Their corresponding polydispersity was slightly higher (see Fig. 3b and Table 1) for that final nanomaterial. Here, the volume distribution was chosen to show the closer measured scattering from the compared samples. Those parameters changes were directly attributed to the presence of bulky PPz molecules attached to the silica nanoparticles surface.

**Table 1:** Characterization data of the prepared nanoparticles (BET specific surface values, pore volumes, pore sizes calculated from the N_2_ adsorption–desorption isotherms, and particle diameter of the nanoparticles)

MSNs	*S*_BET_ m^2^ g^−1^	Pore vol.^a^ cm^3^ g^−1^	Pore size^a^ nm	*D*_h_ b nm (PdI)
MSN0	998	0.93	2.9	108 ± 50 (0.221)
MSN1-SAF-AZ	762	0.74	2.8	110 ± 38 (0.403)
MSN1-SAF-PPz	632	0.58	—	144 ± 54 (0.226)

a Pore volumes and pore sizes are associated with intraparticles mesopores, estimated by BJH model.

b Hydrodynamic diameters of the nanoparticles measured by DLS (average by volume of 6 independent measurements).

To confirm this assumption, the material MSN1-SAF-PPz was analyzed by energy-disperse X-ray spectroscopy (TEM-EDX) and a substantial coverage of PPz attached to the outer silica surface was clearly visualized (see TEM image of MSN1-SAF-PPz in Fig. 3c were the polymer is marked by arrows). Remarkably, all the expected elements (Si, O, Gd, P, N and S) were well distributed and localized on the mesoporous surface (see mapping images in Fig. 3d). For the measurement, a uranium-free staining contrast solution containing Gd, among other non-radioactive elements, was used to enhance the organic side contrast against the inorganic silica-based nanoparticles. It is worth noting that although the polymer was grafted *via* copper-catalysed click reaction, no characteristic signal from this metal was detected in the final nanomaterial, an important consideration for the bioapplication of the nanosystems.

Additionally, the characteristic structural properties of the hybrid nanoparticles were characterized after each loading and functionalization steps. Surface area, pore volume and pore size were obtained from their corresponding N_2_ adsorption–desorption analysis (see structural parameters in Table 1). Typically, isotherms type IV presenting an adsorption step at 0.1–0.3 *P*/*P*_0_ were obtained (see isotherms related to safranin-loaded and PPz-functionalized nanoparticles compared to the calcined control material in Fig. 3a). MSN0 showed typical structural parameters of mesoporous silica nanoparticles type MCM-41 (surface area of *ca.* 1000 m^2^ g^−1^, pore volume of 0.93 cm^3^ g^−1^, and pore size of 2.9 nm). In comparison, the nanomaterials MSN1-SAF-AZ presented smaller surface areas and pore volumes (762 m^2^ g^−1^ and 0.74 cm^3^ g^−1^) calculated by BET and BJH models. These features were consistent with previous reported materials with partially filled mesopores and functionalized surfaces with short moieties,^33,34^ such as the here used azide moieties. Similar pore sizes (2.8 nm) compared with bare calcined MSN0 was obtained for this intermediate material (see BJH pore size distribution curve in Fig. 3a). As expected, the final nanomaterials functionalized with polyphosphazene (MSN1-SAF-PPz) showed a characteristic decrease in surface area, *ca.* 100 m^2^ g^−1^, which further confirms, along with above previous characterization results, the presence of grafted polymer on the external silica surface. The pore size values for the MSN1-SAF-PPz nanomaterial was not considered (not shown in the table) due to classic analysis miscalculations in fully closed pores.^34^

In a further step, the content of polymer (PPz) and cargo (SAF) was determined by thermogravimetric analysis (see Fig. SI-4a in ESI†). A weight loss assigned to the cargo and the azide moieties for materials MSN1-SAF-AZ was 4.48% (in agreement with the loss of surface area), and 10.33% organic weight loss was associated with the polymer in the nanomaterial MSN1-SAF-PPz, and in comparison with calcined MSN0. These contents were similar to other gated mesoporous silica nanocarriers reported in literature.^35^ Moreover, the *ζ*-potential measurements of the nanoparticles provided further information about the proper functionalization at each preparation step. As expected, the surface properties of the undecorated MSN0 commonly covered by –OH groups was highly negative (−48 mV) and in agreement with this type of nanoparticles.^19^ Similarly, the nanoparticles loaded with azide exhibited a positive increase of the *ζ*-potential which may be due to the effective grafting step. Surprisingly, after the polymer grafting step the surface charge was not significantly affected. In general, the PPz improved the dispersion of these silica-based nanocarriers over time, observed during the samples preparation in aqueous solution.

### 2.4. Cargo delivery from the dual temperature and pH responsive hybrid nanosystem

The dual gate-like behavior of the nanoparticles MSN1-SAF-PPz was studied by following the release of the cargo (safranin O) under different conditions of temperature and pH. Initially, in order to study the thermoresponsive role of the Jeffamine-based polyphosphazene on the gate nanosystem, the dye release from MSN1-SAF-PPz was carried out at cold temperatures (at 5 °C, below LCST_PPz_) and around human body temperature (at 37 °C, above LCST_PPz_), both under neutral pH 7.4 conditions, simulating physiological blood pH. The release studies were monitored for 24 h (1440 min).

**Fig. 4:**
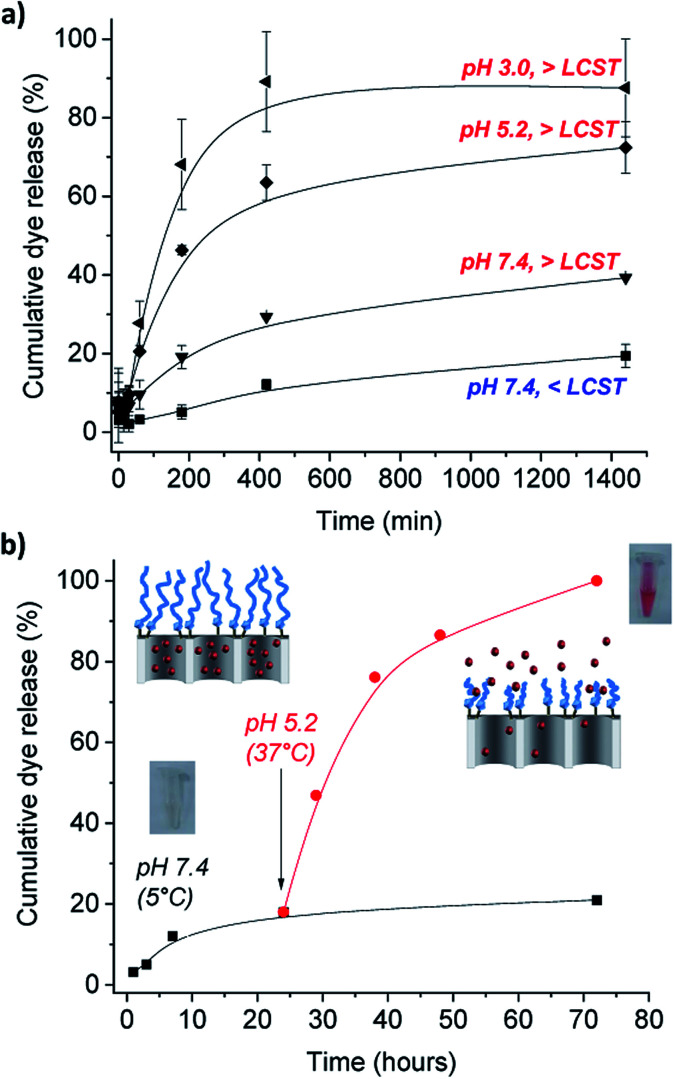
Cumulative release profiles of (a) safranin O (SAF) from PBS suspensions of MSN1-SAF-PPz nanoparticles at pH 7.4 (dye absorbance measured at 520 nm) below (<LCST = 5 °C) and above (>LCST = 37 °C) the PPz LCST, showing the temperature effect, and release profiles at acidic pHs 5.2 and 3.0 above the LCST (at 37 °C) of the dual pH and temperature responsive gates based on Jeffamine® M-2005 derived polyphosphazene (PPz) functionalized on the silica surface. Error bars expressed as *σ* from three independent experiments; (b) cumulative partial dye release from PBS suspensions of MSN1-SAF-PPz at cold and neutral conditions for 24 h and suddenly acidic decrease of pH and temperature rise above LCST_PPz_, at 37 °C, for 48 h more. Inset: colourless dye release solution collected after of 24 hours at pH 7.4 and pink solution from the dye released at after 48 hours at pH 5.2.

Of importance was also the performance of the nanomaterial in acidic conditions to simulate the cells internal lysosomal pH medium. Following the same procedure that with temperature changes, the pH effect was assessed by performing the release studies in phosphate buffer solutions at slightly acidic pH 5.2, typically from late endosomes and also of tumor microenvironments, and at more acidic aqueous solution (prepared at pH 3.0).^36^ An obvious marked different response was observed from the release profiles showed in Fig. 4a. At neutral pH (which is typical from healthy organs) and temperatures below PPz LCST showed the lowest amount of released cargo (*ca.* 10 after 7 h), as the molecular gates are effectively closed due to the expanded conformation of the PPz. Upon increasing the temperature to above the LCST, release was increased to a modest 27%, due to collapse of the PPz on the MSN surface. However, a much faster dye release was observed when both effects of pH and temperature were involved, yielding to a cumulative dye released of 58 and 82% after 7 h that kept steady after 24 h of 72% and 88% cargo release measured under acidic conditions for pH 5.2, and pH 3.0, at above body temperatures (see complete UV-Vis spectra from dye released in ESI, Fig. SI-5†). The faster cargo release with the higher temperatures and lower pHs is caused by collapsing of the polymer attached to the silica surface unblocking the pores. While polyphosphazenes are known to degrade at low pH values over a period of hours or days,^21^ the rapid release of dye (in the first minutes) observed cannot be explained by this. It is however known that polyaminophosphazenes become highly protonated and hence, cationic at lower pH values^23,37^ and it is possible that this behavior induces conformational changes in the polymer, leading to the clearly detected increased release. Alternatively, it is possible that the positive charge of the PPz at lower pH enhances expulsion of the charged dye from the pore. Future studies will be required to confirm this mechanism.

Furthermore, this potential close-open gate mechanism of the hybrid nanomaterial was investigated by suddenly changing the conditions of pH and temperature, and following the partial release studies of the dye from the pores (Fig. 4b). Similarly, a poor release was detected at neutral pH and cold temperatures for 24 h, assuming the gate is closed due to the stretched confirmation of the polymer under these conditions (see Fig. 4b). A marked dye release was immediately detected after 48 more hours at pH 5.2 and 37 °C, and easily observed by the naked-eye (see inset images of the release cargo in Fig. 4b). This result was in agreement with the previous triggered response of the functionalized MSN1-SAF-PPz at acidic conditions and temperatures above its LCST (here at 37 °C). Significantly, the ability to minimize uncontrolled release doses for future biological applications was solved here by using the gated nanomaterials, which progressive release profile over longer periods of time (up to 72 h) was demonstrated (see Fig. 4b, pH 7.4).

It is worthy to remark the advantages of this pH and temperature controlled release in terms of safety. Although the release studies were performed with the dye to facilitate the experimental set up, for lung tumor therapies, a chemotherapeutic drug such as camptothecin will be encapsulated. As it is widely known, the main problem of chemotherapy is the production of side effects due to the systemic biodistribution of the drug. Although our nanoparticles are not actively vectored to tumors, thanks to the enhanced permeability and retention effect, they will be preferentially accumulated in tumor microenvironment. Moreover, since the release of the drug will only take place upon pH decrease, both factors will contribute to the decrease of the side effects that will enable the increase of the administered dose, thus allowing the efficacy of the treatment.

Therefore, after having demonstrated the well performing dual responsive behavior of the prepared nanoparticles functionalized with PPz and loaded dye, the application for therapeutic delivering purposes within cancer cells was attempted. For this, a similar nanomaterial but loaded with the chemotherapeutic drug camptothecin (CPT), MSN2-CPT-PPz, was prepared and tested directly on models of healthy and tumor lung cells. This selected payload is a well-known chemotherapeutic agent whose hydrophobic character makes this drug difficult to use. Hence, the possibility to transport it within the pores of mesoporous silica nanoparticles can decrease its solubility problems in water,^38^ while its selective intracellular distribution by means of the stimuli-responsive polyphosphazene gatekeepers could contribute strongly to avoid further unwanted side effects. This new material called MSN2-CPT-PPz showed similar features than its partner loaded with safranin O. The nanomaterial MSN2-CPT-PPz presented similar surface areas and pore volumes (451 m^2^ g^−1^ and 0.31 cm^3^ g^−1^, (see corresponding N_2_ adsorption–desorption isotherms and BJH pore size distribution curves for this material in Fig SI-3, ESI†). The content of drug and polymer was also determined by thermogravimetric analysis (see TGA spectra in Fig. SI-4 in ESI†), amounted to similar functionalization of polymer on the silica surface (*ca.* 12%), but higher cargo loading (*ca.* 28%) in comparison to MSN1-SAF-PPz, but still within the loadings orders reported in literature.^34,35^ That difference between uploading could be attributed to the different hydrophobic character of the selected cargo molecules which may change the extent of incorporation within the mesopores during the uploading process.

### 2.5. Cargo delivery from the responsive hybrid nanosystem inside intracellular media

**Fig. 5:**
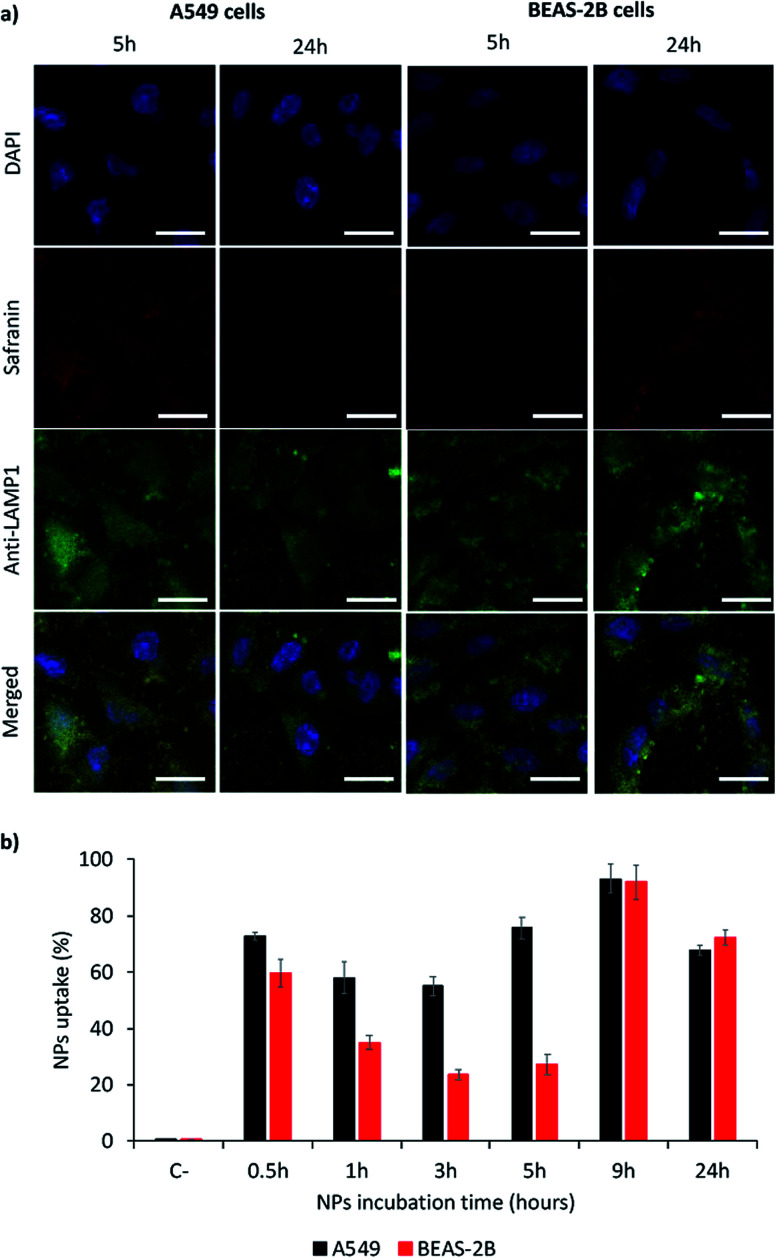
Uptake study of MSN1-SAF-PPz in tumor (A549) and epithelia (BEAS-2B) lung model cells. (a) Qualitative study by confocal micrographies of nanoparticles after their incubation during short (5 h) and long (24 h) times with cells. Blue signal: nuclei staining by DAPI; red signal: safranin; green signal: lysosomes labelling using anti-LAMP1 antibody. Scale bar = 25 μm. (b) Quantitative study by flow cytometry of MSN1-SAF-PPz uptake in model cells by quantifying safranin.

Nanoparticles MSN1-SAF-PPz were selected as models for the study of their internalization in models of lung cancer cells (A549), as target cells, and of lung epithelial cells (BEAS-2B), as non-target cells. These responsive hybrid nanosystems were *in vitro* incubated with both cell lines, during different times, to determine a kinetics of the cell internalization and their uptake was studied by confocal analysis (qualitatively) as well as for flow cytometry analysis (quantitatively). As shown in Fig. 5a (also complete times for A549 cells shown in Fig. SI-7† and for BEAS-2B cells in Fig. SI-8†), safranin uptake took place for both cell lines, indicating that MSN1-SAF-PPz nanoparticles were able to penetrate tumor and healthy cells. However, while the uptake was very fast for A549 cells, achieving around 60% of cells with internalized nanoparticles at all time-points studied (Fig. 5b), for BEAS-2B it took around 9 h to achieve a marked uptake, of around 80%. It is noteworthy to remark that at very short incubation-time points, the uptake seems very high for both cell lines tested, according to cytometry data. However, these results could be attributed to an attachment of the nanoparticles on the cell surface, specifically for epithelial lung cells, since, at longer times, the percentage of cells that contain safranin internalized decreased notable at medium times. This effect could be also attributed to the rapid elimination of a fraction of the nanoparticles by BEAS-2B cells at intermediate times.

Another factor to be taken into account is the subcellular localization of the internalized nanoparticles.^39^ To study this effect, we added a marker for lysosomes (anti-LAM1 antibody). The accumulation in lysosomes is important for lysosomes targeting, and could also be advantageous in terms of designing novel therapies for potential lysosomal storages diseases.^40^ This could be justified by a rapid intracellular trafficking of the nanoparticles, expected to be endocytosed by cells to achieve the cell uptake.^39^ Relating these results with viability studies, we may suggest that lysosomal accumulation of the cargo could be advantageous in terms of elimination of the materials after their pharmacological activity which could be beneficial in terms of avoiding toxicities caused by accumulation of nanosystems.

**Fig. 6:**
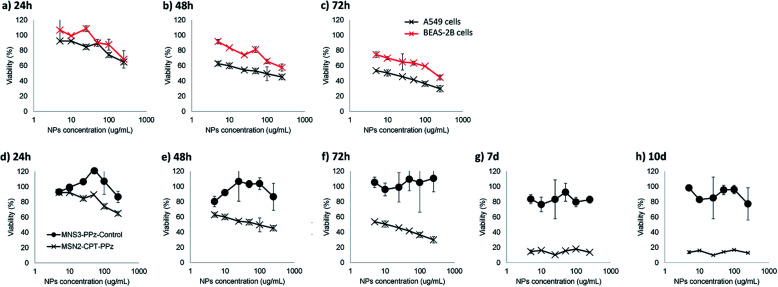
*In vitro* viability studies based on MTT metabolic assays of: (a), (b) and (c) MSN2-CPT-PPz incubated with lung epithelial (BEAS-2B) and lung tumor (A549) cells, for different times, from (d) to (h) MSN3-PPz-control and MSN2-CPT-PPz nanoparticles incubated with A549 lung tumor cells.

Accordingly, our further measurements regarding potential cytotoxicity of the prepared nanoparticles would enable us to confirm that, a part from the nanoparticles being rapidly co-localized within lysosomes, they could represent a promising lung cancer controlled cytotoxic treatment. Camptothecin, being a topoisomerase I inhibitor, is used thanks to its antineoplastic activity as cancer chemotherapeutic drug. However, its use is limited due to the production of severe side effects in healthy growing cells. For this reason, and due to its poor water solubility, its encapsulation in targeted nanocarriers, as here within mesoporous silica nanoparticles, is strongly recommended to enhance future therapies safety. Hence, we studied the cytotoxicity produced in target (A549 lung tumor) and non-target (BEAS-2B lung epithelial) cells. In addition, we also studied the possible cytotoxicity of bare nanoparticles (MSN3-PPz-control) as well as that from camptothecin-loaded nanoparticles (MSN2-CPT-PPz) at different time-points (Fig. 6 shows the most MTT relevant results).

As shown in Fig. 6a–c, MSN2-CPT-PPz produced a concentration and time-dependent toxicity in A549 lung tumor model cells. In addition, healthy epithelial lung cell mortality was lower at all time points and in all concentrations tested, as compared with lung cancer cells, which enables us to confirm that MSN2-CPT-PPz cytotoxicity was cell-type dependent. In a further study to confirm that toxicity came from the active drug (camptothecin) and not from the material used as the carrier, we also compared the lung cancer cells viability after their incubation with MSN2-CPT-PPz and with MSN3-PPz-control nanoparticles during different times. As it is clearly plotted in Fig. 6d–h, non-loaded nanoparticles did not produce cytotoxicity in lung tumor cells up to 10 days. Therefore, the cytotoxic effect was attributed directly to the encapsulated chemotherapeutic drug.

## 3. Experimental

### 3.1. Chemicals

Safranin O (≥85%), sodium ascorbate (99%), tetraethyl orthosilicate (TEOS, 98%), lithium bis(trimethylsilyl)amide (LiN(SiMe_3_)_2_, 97%), phosphorous trichloride (PCl_3_, 99%), dichlorotriphenylphosphorane (Ph_3_PCl_2_, 95%), were purchased from Sigma Aldrich. Cetyltrimethylammonium bromide (CTAB, >99%) was purchased from VWR. Triethylamine (Et_3_N) from Merck was distilled and dried over molecular sieves (3 Å). Sodium hydroxide (98%) and copper(ii) sulfate pentahydrate (98.5%) were purchased from J. T. Barker. 3-Azidopropyl triethoxysilane (97%), camptothecin (>98%) and 2-propynylamine (97%) were purchased from fluorochem. Polyetheramine copolymer (PEO-PPO-NH_2_), sold under trade name Jeffamine® M-2005, with nominal molecular weight of 2000 g mol^−1^, was purchased from Huntsman Performance Products. Deuterated chloroform (CDCl_3_), (99.8%, Sigma Aldrich) was used for NMR measurements. Uranyless EM Stain (contrast stain solution for TEM) was purchased from EMS. Solvents were purchased from VWR and Alfa Aesar, and used as received if not stated otherwise. Anti-LAMP1 rabbit primary antibody and goat anti-rabbit secondary antibody IgG H&L (Alexa Fluor® 488) were purchased from Abcam. DAPI was purchased from Sigma-Aldrich.

### 3.2. Cell lines

A549 (ATCC CCL-185) and BEAS-2B (ATCC CRL-9609) cell lines were maintained in DMEM supplemented with 10% (v/v) heat inactivated fetal bovine serum (FBS, research grade, HyClone™), 100 units mL^−1^ penicillin G, 100 μg mL^−1^ streptomycin, and 2 mmol L^−1^l-glutamine. All cells were cultured at 37 °C, under a 5% CO_2_/95% air atmosphere until 90% confluence before starting transfections.

### 3.3. Characterization techniques

^1^H-NMR and ^31^P-NMR spectra were recorded with 300 and 121 MHz, respectively, using a Bruker® Advance 300 spectrometer. 18 MΩ ultra pure water (Milli-Q water) was obtained from Millipore device with a Millipak® express 40 filter (0.22 μm pore size). BET-data were obtained from a TriStar II 3020 V1.01 was employed with N_2_ as adsorptive in an analysis bath at 77.30 K. Thermogravimetric analyses were carried out with a TGA/PerkinElmer Q5000, with platinum pans and measurements between 50 and 900 °C, heating rate of 10 °C min^−1^ under nitrogen (25 mL min^−1^). Transmission electron microscopy (TEM) images were obtained and elemental mapping analyses (TEM-EDX) were performed with a Jeol JEM-2200FS microscope at 200 kV. For TEM samples preparation, Milli-Q was used as dispersant (0.1 mg mL^−1^), and sonication for 15 minutes to disperse the sample. Nanoparticles were placed on 300 mesh copper grids coated with a holey carbon film. High-resolution TEM images were recorded by means of zero-loss filtering, using an in-column Ω-filter. A Zetasizer Nano ZSP (Malvern Instruments) was used to obtain the hydrodynamic diameter (*D*_h_) and zeta potential, by dynamic light scattering (DLS) of nanoparticles in Milli-Q water (1 mg mL^−1^). For each measurement, 6 runs were performed. Lower critical solution temperatures (LCST) were determined by monitoring the optical transmittance at 500 nm of 1 mg mL^−1^ of polymers in aqueous phosphate buffered solution (PBS, pH 7.4 and 5.2) and acidic aqueous solution (pH 3.0) at temperatures ranging from 10 °C to 45 °C (1 °C min^−1^), using a Cary UV 100 UV-Visible spectrometer equipped with a temperature programmer. The LCST was determined at 50% transmittance value (sudden slope change in the transmittance curve). UV-Vis spectra measurements were performed using a PerkinElmer Lambda 35 UV-Visible spectrometer.

### 3.4. Synthesis

#### 3.4.1. Jeffamine-based polyphosphazene (PPz)

In a first step, the precursor poly(dichloro)phosphazene [NPCl_2_]_*n*_ (*n* ∼ 50) was prepared, according to phosphine-mediated polymerization already reported by our group.^31^ Dichlorotriphenylphosphorane (43.8 mg, 0.13 mmol, 1 eq.) was dissolved in anhydrous DCM (0.5 mL) and mixed with a solution of Cl_3_P=N–SiMe_3_ (1.68 g, 6.30 mmol, 50 eq.) in anhydrous DCM (0.5 mL), stirred for 24 h in a glovebox. This polymer solution was used directly in the next step reaction without further purification. In a second step, the macromolecular substitution was started by adding Et_3_N (0.44 mL, 3.16 mmol) and 2-propynylamine (0.21 mL, 3.16 mmol) in THF (2 mL) dropwise to the previously synthetized polymer solution. The solution was stirred for 24 h at room temperature. Then, more Et_3_N (0.9 mL, 6.46 mmol) and Jeffamine® M-2005 (12.61 g, 6.31 mmol) dissolved in THF (10 mL) were added. After 24 h, 2-propynylamine (1 mL, 15.61 mmol) and Et_3_N (2.2 mL, 15.78 mmol) were added in excess. Once the complete substitution of all chlorine atoms was confirmed by ^31^P NMR spectroscopy, the polymer solution was filtered and the solvent removed under vacuum. The polymer was first purified in ice water and in cooled EtOH for 24 h each (dialysis membranes 6–8 kDa cut-off), and then again for 120 h in ice water (12–14 kDa cut-off). Afterwards, the water was removed by evaporation under vacuum, and further dried to yield a viscous liquid polymer PPz (6.22 g, 46.9%, ratio Jeffamine® M-2005: 2-propynylamine 1 : 2). ^1^H NMR (300 MHz, CDCl_3_, *δ*): 3.35–3.62 (108H, –OCH_2_, –CH_2_–C≡C), 2.22 (s, 2H, CH≡C–), 1.10 (m, 87H, –CH_3_ of PPO) ppm; ^31^P NMR (121 MHz, CDCl_3_, *δ*/ppm): 0.07 (–N=PR_2_–), 17.70 (–N=PPh_3_) ppm.

#### 3.4.2. Mesoporous silica nanoparticles (MSN0)

The synthetic procedure to prepare mesoporous silica nanoparticles (MSNs) was followed according with previous reports.^33^ Surfactant CTAB (1 g, 2.74 mmol) was dissolved in 480 mL of pure water, followed by the addition of NaOH (3.5 mL, 2 M). Then, TEOS (5 mL, 22.4 mmol) was added dropwise to the surfactant mixture at 80 °C while stirring. After 2 h, the white precipitated obtained was isolated by centrifugation and washed vigorously with deionized water until neutral pH. The as-synthesized nanoparticles were dried at 60 °C for 12 h and finally, the surfactant was removed by calcination at 550 °C for 5 h obtaining the calcined white powder-like material MSN0.

#### 3.4.3. Azide-functionalized mesoporous silica nanoparticles loaded with safranin O (MSN1-SAF-AZ)

Safranin O (100 mg, 0.29 mmol) and previously calcined mesoporous silica nanoparticles MSN0 (350 mg) were suspended in CH_3_CN (35 mL). Then, the suspension was heated to 110 °C to remove adsorbed water (10 mL) by azeotropic distillation under nitrogen atmosphere using a Dean–Stark set-up. After stirring the mixture for 24 h at room temperature, 3-azidopropyltriethoxysilane (0.4 mL, 1.5 mmol) was added and the mixture stirred for another 6 h. The resulting mixture was centrifuged and washed with CH_3_CN (12 mL × 2). The final nanoparticles were dried at 40 °C in a vacuum oven for 12 h, yielding to MSN1-SAF-AZ as red powder-like material.

#### 3.4.4. PPz-functionalized mesoporous silica nanoparticles loaded with safranin O (MSN1-SAF-PPz)

With the aim of attaching the Jeffamine-based polyphosphazene *via* azide–alkyne Huisgen cycloaddition, the material MSN1-SAF-AZ (100 mg) was dispersed in DMSO : H_2_O mixture (5 mL : 4.75 mL) and sodium ascorbate (4 mg) and CuSO_4_·5H_2_O aqueous solution (20 μL, 1 mM) were added. Afterwards, the polymer was added (0.25 mL from 2 mg mL^−1^ aqueous solution of PPz, 4.5 × 10^−9^ mol, equal to 0.4 eq. of corresponding triple bonds) leading to a 1 : 1 ratio of both solvents, and the reaction mixture was stirred for 24 h at room temperature. Finally, the resulting nanoparticles were centrifuged and washed with H_2_O (15 mL × 4) and CH_3_CN (15 mL × 2) and dried under vacuum at 35 °C for 12 h, to yield the pink-red powder MSN1-SAF-PPz.

#### 3.4.5. Azide grafted mesoporous silica nanoparticles loaded with anticancer drug camptothecin (MSN2-CPT-AZ)

Cytotoxic drug camptothecin (140 mg, 0.40 mmol) and previously calcined mesoporous silica nanoparticles MSN0 (250 mg) were suspended in CH_3_CN : MeOH (1 : 1, 60 mL) under nitrogen atmosphere. After stirring the mixture for 24 h at room temperature, 3-azidopropyltriethoxysilane (0.27 mL, 1.1 mmol) was added and the mixture stirred for another 6 h. The resulting mixture was centrifuged and washed with CH_3_CN (10 mL × 2). The final nanoparticles MSN2-CPT-AZ were dried under vacuum at 35 °C for 12 h, as yellowish powder material.

#### 3.4.6. Synthesis of PPz grafted nanoparticles loaded with anticancer drug camptothecin (MSN2-CPT-PPz)

Following same procedure as for preparing MSN1-SAF-PPz, the material MSN2-CPT-AZ (100 mg) was dispersed in DMSO : H_2_O (5 mL : 4.75 mL) and sodium ascorbate (4 mg) and CuSO_4_·5H_2_O aqueous solution (20 μL, 1 mM) were added. Afterwards, the polymer was added (0.25 mL from 2 mg mL^−1^ aqueous solution of PPz, 4.5 × 10^−9^ mol, equal to 0.4 eq. of corresponding triple bonds), leading to a 1 : 1 ratio of both solvents, and the reaction mixture was stirred for 24 h at room temperature. Finally, the resulting nanoparticles were centrifuged and washed with H_2_O (15 mL × 4) and CH_3_CN (15 mL × 2) and dried under vacuum at 35 °C for 12 h, obtaining MSN2-CPT-PPz as yellowish powder-like material.

#### 3.4.7. Synthesis of non-loaded nanoparticles functionalized with PPz (MSN3-PPz-control)

An extra material was prepared following the same procedure as for nanoparticles MSN2-CPT-PPz but without any cargo, grafting PPz on the silica surface, yielding to white powder-like material MSN3-PPz-control.

### 3.5. Cargo release studies

In a typical release experiment, prepared solid MSN1-SAF-PPz (5 mg) was suspended in 12.5 mL of PBS solutions at pH 7.4 and pH 5.2, and acidic solution of pH. Aliquots of 1 mL were collected and filtered (to remove the nanoparticles) at certain times during 24 h. The absorbance of safranin O at 520 nm was measured for 24 h (1440 min). Partial dye release studies were also followed only with MSN1-SAF-PPz nanoparticles, as a function of temperature variation at 5 °C (<LCST) and at 37 °C (>LCST). For this experiment, 5 mg of nanoparticles were suspended in 12.5 mL of PBS at pH 7.4 and stirred for 24 h, while aliquots of 1.0 mL were collected and centrifuged. The absorbance of safranin O was measured at 520 nm. After 24 h, the temperature was increased to 37 °C. The same samples collecting and measuring procedure was followed for 48 h. The whole experiment was performed over 72 h in total.

### 3.6. Nanoparticles cellular uptake studies

Cells were seeded either in a 96-well plate or in glass copper slices placed in a 6-well plate, previously coated with 0.1% gelatin solution, at 90% confluence, 24 h before starting the experiment. MSN1-SAF-PPz was incubated with cells for different experimental times, to determine cell uptake. Nanoparticles uptake, using safranin as reporter (*λ*_excitation_ = 532 nm; *λ*_emission_ = 560 nm), were determined. Qualitative studies were carried out by Leica TCS SP8 laser-scanning confocal spectral microscope (Leica Microsystems Heidelberg, Mannheim, Germany) with Argon and HeNe lasers attached to a Leica DMi8 S Platform inverted microscope. For visualization of the nanoparticles uptake, images were acquired using an APO 40× objective lens. Numerical aperture 1.4, 405, 488 and 528 nm laser lines, acoustic beam splitter, emission detected in the range of 410–430, 500–520, 535–580 and the confocal pinhole set at 1 Airy units. Nuclei were stained by 5 min incubation with DAPI. Immunofluorescence was performed to label lysosomes through LAMP1 staining and further mounting the copper slices in a glass slide. Flow cytometry (NovoCyte, ACEA Bioscience) was used to quantify uptake efficiency. Briefly, at the end of the experiment, cells were trypsinized and fixed with 1% paraformaldehyde. At least 2000 cells were analyzed for each well. Results correspond to the mean ± standard deviation of at least three independent experiments, each consisting on an experiment triplicate.

### 3.7. *In vitro* viability studies

Cells were seeded in a 96-well plate at 90% confluence, 24 h before starting the experiment. Cells were incubated with increasing amounts of the different kinds of nanoparticles tested, at increasing concentrations. Non-treated cells were used as negative controls (100% viability). *In vitro* cell viability was evaluated through quantification of cell metabolic activity, by using MTT colorimetric assay. Briefly, nanoparticles were incubated for 48 h with cells. After this time, media were removed and replaced with 0.5 mg mL^−1^ MTT in complete media and further incubated for around 3 h. Following, media were removed and formazan crystals were dissolved in 100 mL DMSO. Absorbance was quantified at 570 nm, using a plate reader (SpectraMax M5, Molecular Devices). Results are expressed as percentage of viable cells related to non-treated cells (100% viability).

## 4. Conclusions

We describe the use of pH and thermoresponsive bottle-brush polyphosphazenes as a gatekeeper for dual responsive mesoporous silica nanoparticles, designed to release the uploaded cargo at acidic pH conditions and body temperatures. A mesoporous silica nanomaterial MSN1-SAF-PPz was loaded with a selected dye (safranin O, SAF) as a model cargo and covalently decorated with azide units, to facilitate the subsequent functionalization with the PPz *via* azide–alkyne “click” reaction enabling the easy grafting to the silica pores outer surface. A detailed characterization was carried out to ensure the presence of the polymer on the nanoparticles, which was clearly verified. The potential close-open dual response mechanism of the gate hybrid nanosystem was demonstrated, showing negligible release at neutral pH, related to healthy cells, and cold temperatures, which can be selectively opened, leading to rapid payload release under acidic conditions at body temperature (typical of cancer cells). The same type of mesoporous silica nanoparticles functionalized with PPz but not loaded (MSN3-PPz-control) were tested showing positive high biocompatibility, which could open up the potential use *in vitro* and *in vivo*. Therefore, the possible bioapplication of the gated system for on demand controlled delivery of lung cancer treatments was directly translated to *in vitro* studies by the preparation of the same formulation but loaded with chemotherapeutic drug camptothecin, CPT (nanomaterial MSN2-CPT-PPz). We demonstrated that these nanoparticles were able to penetrate cells both tumor and healthy cells, being their toxic effects more pronounced in tumor cells. In addition, it became clear by confocal microscopy that they accumulate in lysosomes, which is an advantageous point for their further use for lysosome targeting and in terms of avoiding toxicity due to accumulation. This was confirmed by the selective cytotoxicity produced only in lung tumor models when camptothecin was encapsulated as a model chemotherapeutic drug in MSN capped with PPz. In summary, we have demonstrated that a novel MSN formulation, based on a biocompatible pH and temperature responsive polyphosphazene-based molecular gate, is able to produce a selective, concentration and time-dependent toxicity to lung tumor cell models when camptothecin is encapsulated. This works brings up the possibility of using a wide range of responsive units onto the polyphosphazene backbone designed for specific release applications, such as controlled antitumor therapeutics, based on gated silica mesoporous nanosystems.

## Conflicts of interest

There are no conflicts to declare.

## Supplementary Material

RA-010-D0RA03210G-s001
