# Supplementary material for: Dual stimuli-responsive polyphosphazene-based molecular gates for controlled drug delivery in lung cancer cells
Source: RSC Adv. 2020 Jul 21;10(46):27305–14. doi: 10.1039/d0ra03210g (PMC9055533; doi:10.1039/d0ra03210g)
Supplement: RA-010-D0RA03210G-s001 [file RA-010-D0RA03210G-s001.pdf]

Electronic Supplementary Material (ESI) for RSC Advances.
This journal is © The Royal Society of Chemistry 2020

Supporting Information

Stimuli responsive polyphosphazene-based molecular gates for controlled drug delivery in lung cancer cells

Yolanda Salinas,[*a,b] *Michael Kneidinger,*[a] *Cristina Fornaguera,*[c] *Salvador Borrós,*[c]

Oliver Brüggemann[a] *and Ian Teasdale*[a,b]

a. Institute of Polymer Chemistry (ICP), Johannes Kepler University Linz, Altenberger Strasse 69, 4040 Linz, Austria.

b. Linz Institute of Technology (LIT), Johannes Kepler University Linz, Altenberger Strasse 69, 4040 Linz, Austria.

c. Grup d'Enginyeria de Materials (GEMAT), Institut Químic de Sarrià (IQS), Universitat Ramon Llull (URL), Via Augusta 390, 08017, Barcelona, Spain.

***Corresponding Author**

E-mail: yolanda.salinas@jku.at (Y.S.)

Synthesis and characterization of the Jeffamine-based polyphosphazene (PPz)

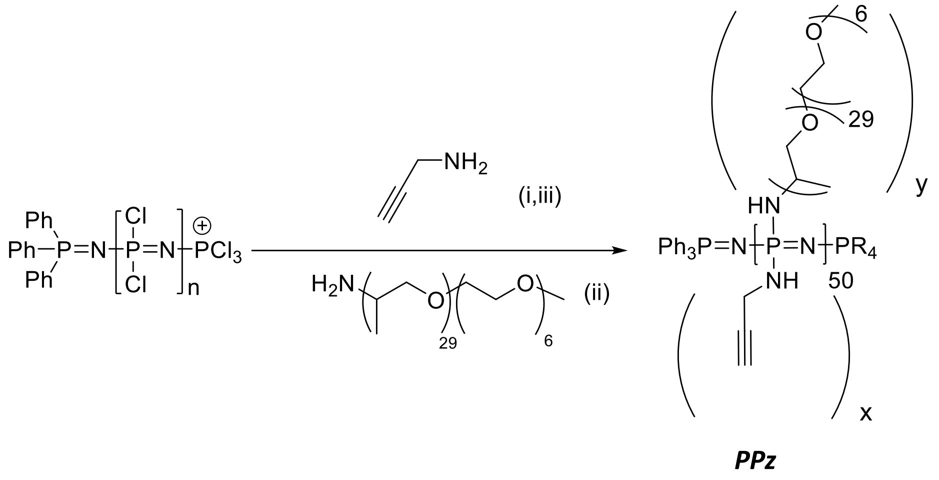

Figure SI-1. Synthesis reaction of Jeffamine-based polyphosphazene (PPz) by macromolecular substitution carried out in three steps: (i) adding propynylamine, then (ii) Jeffamine M-2005 and finally (iii) an excess of propargylamine, n ~ 50. The scheme of the polymer shows combinations of the two different substituents statistically distributed along the backbone in a ratio of propynylamine:Jeffamine M-2005 approximately 2:1 (x = 1.4 and y = 0.6).

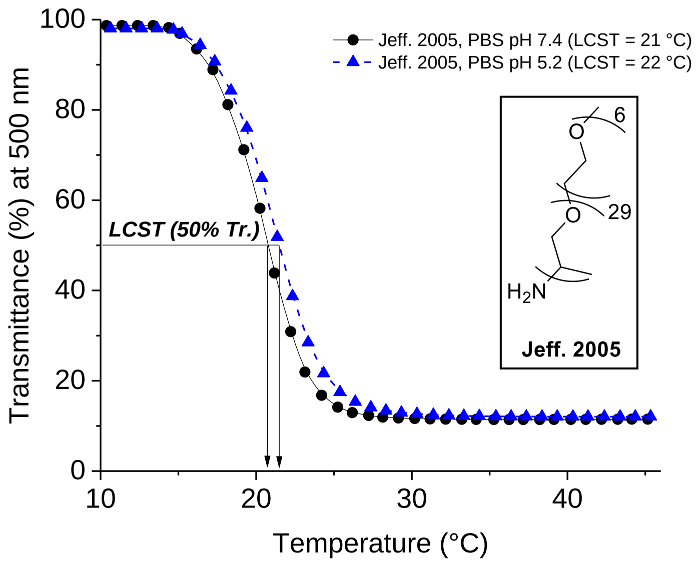

Figure SI-2. Lower critical solution temperature (LCST) of Jeffamine M-2005 (Jeff. 2005) determined by transmittance measurements with increasing temperature at 500 nm for 1 mg mL^{-1} of polymer, as purchased, in phosphate buffer solution at pH 7.4 and 5.2. Inset: chemical structure of the polymer.

Characterization of the hybrid nanomaterials:

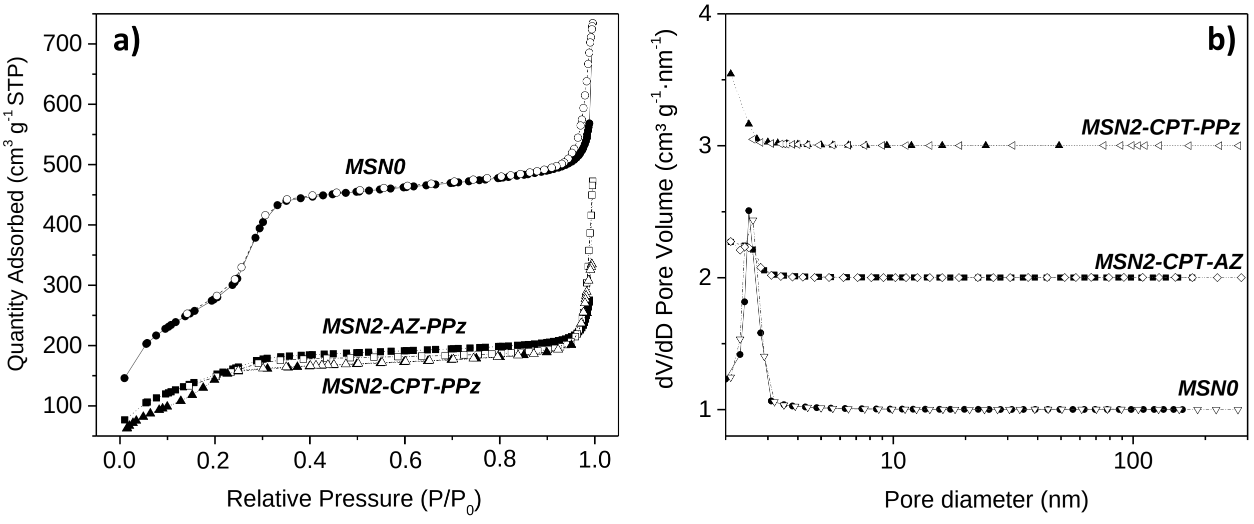

Figure SI-3. a) Nitrogen adsorption-desorption isotherms and b) BJH pore size distribution curves of materials **MSN0**, **MSN2-CPT-AZ** and **MSN2-CPT-PPz**, full symbols and empty symbols stand for adsorption and desorption respectively.

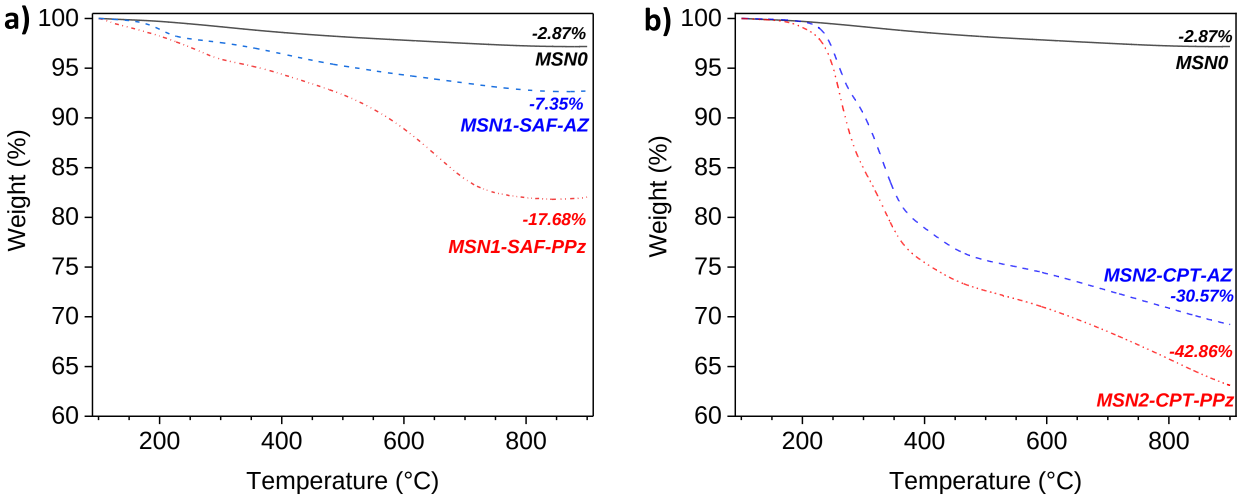

Figure SI-4. Thermogravimetric analysis of all prepared nanomaterials **MSN0**, **MSN1-SAF-AZ**, **MSN1-SAF-PPz**, **MSN2-CPT-AZ** and **MSN2-CPT-PPz**

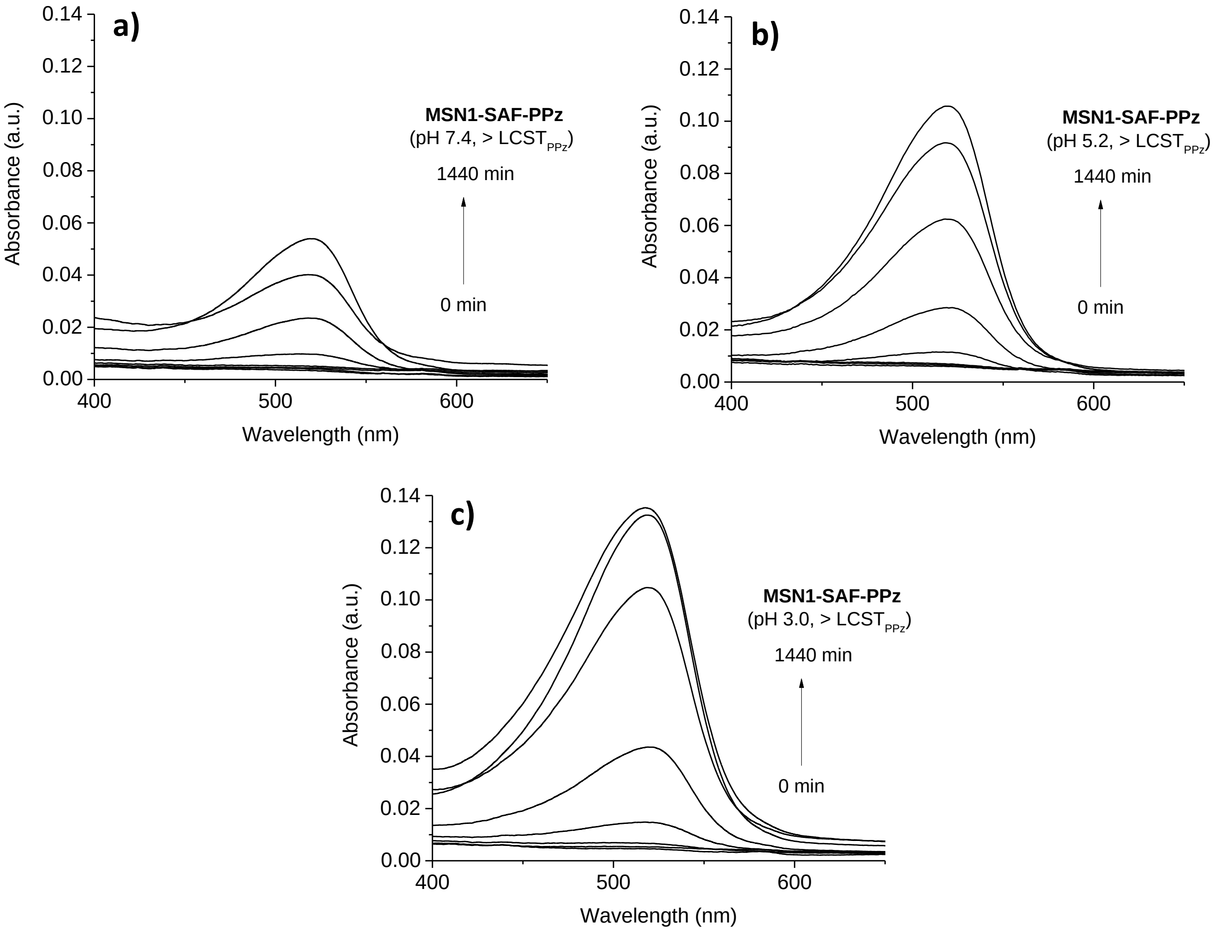

Figure SI-5. UV-Vis spectra of the released safranin O in from the collapsed PPz-capped MSNs (MSN1-SAF-PPz) at pH 7.4 (a), 5.2 (b) and 3.0 (c) during 1440 min at 37 °C (above $LCST_{PPZ}$).

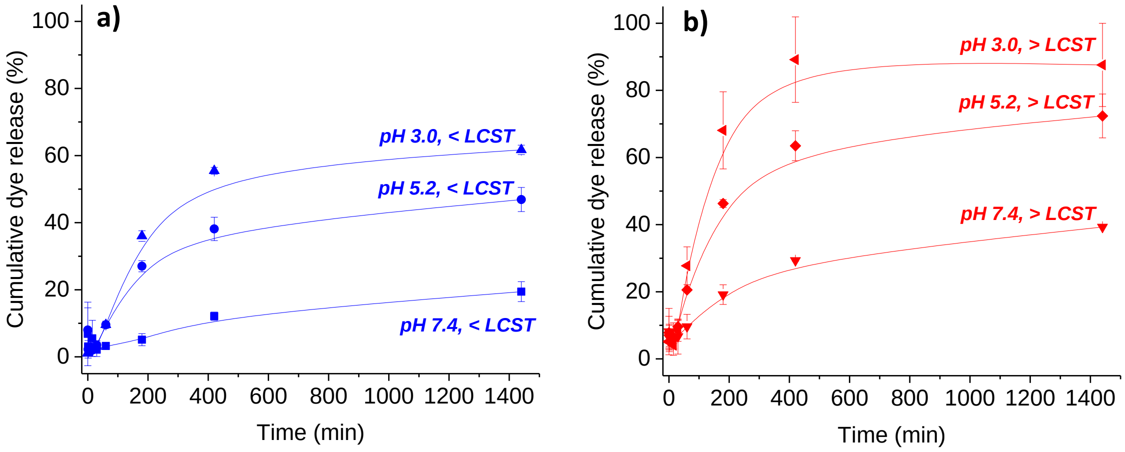

Figure SI-6. Cumulative dye release (%) of Safranin O at 520 nm in aqueous buffered suspensions from PPz-capped mesoporous silica nanoparticles (MSN1-SAF-PPz) at pH 7.4, 5.2 and 3.0 during 1440 min, at a) temperature below and b) above $LCST_{PPz}$ (at 5 °C and 37 °C, respectively).

Cell uptake studies of nanoparticles

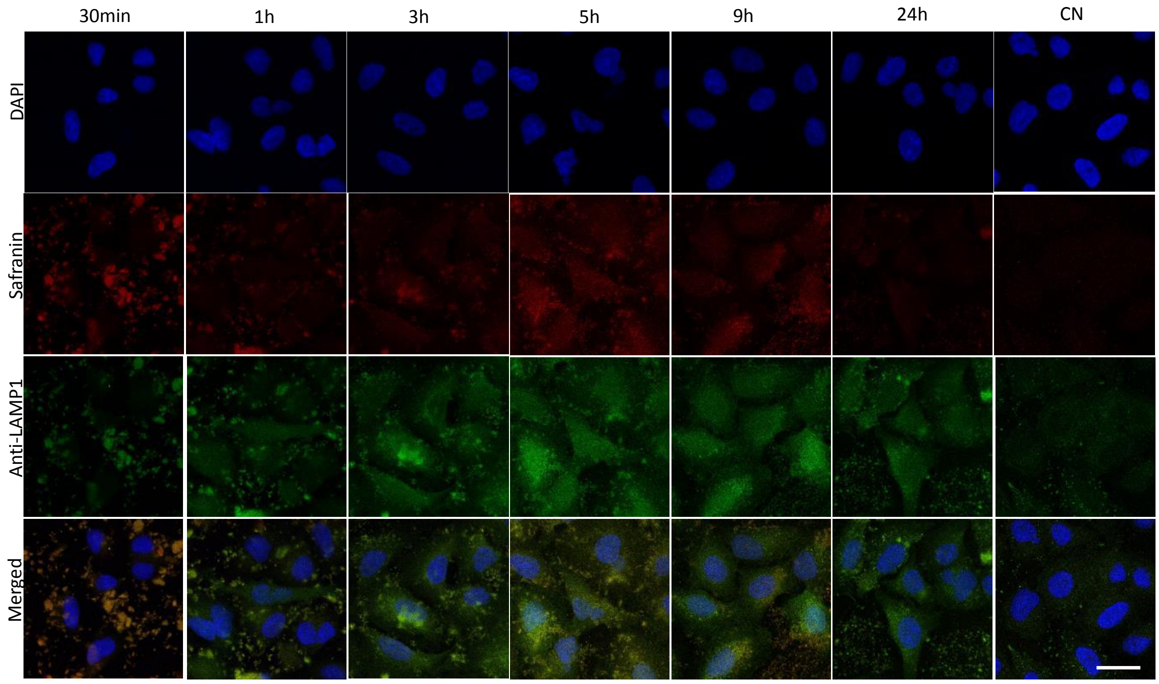

Figure SI-7. Accumulation of the dye safranin O in A549 lung adenocarcinoma cells after different time incubation of cells with nanoparticles loaded with safranin. In addition, nuclei were stained (DAPI), as well as lysosomes (anti-LAMP1 antibody), and all signals were co-localized (merged). Scale bar = 25 μm.

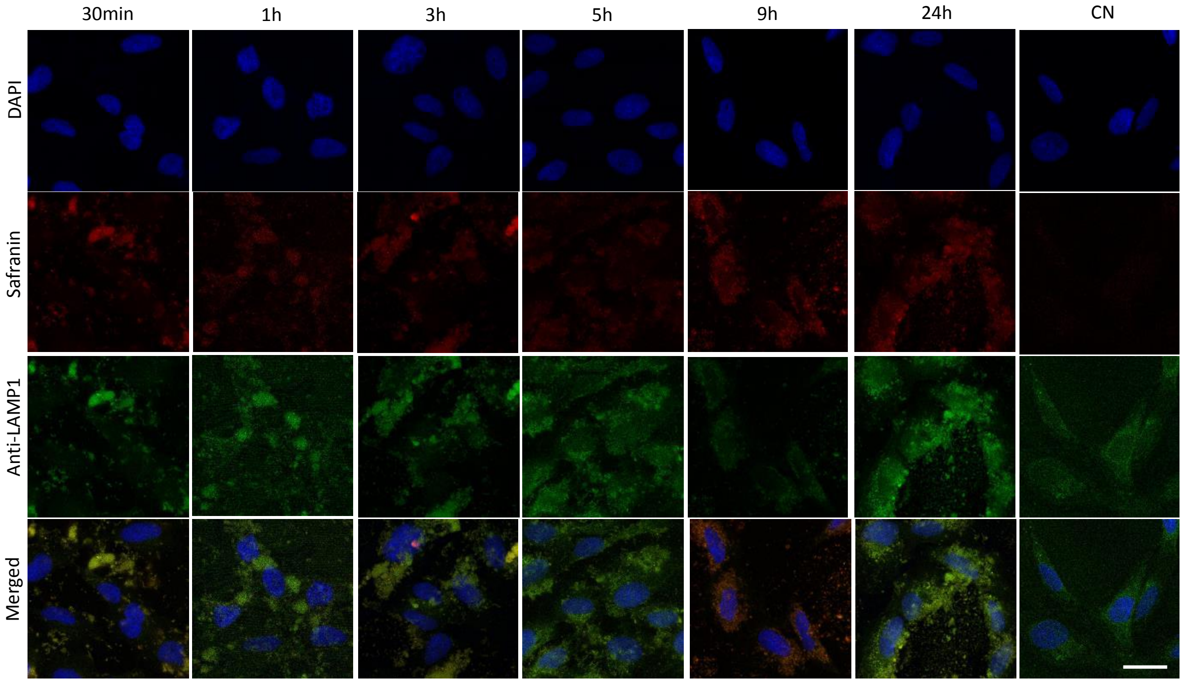

Figure SI-8. Accumulation of the dye safranin O in BEAS-2B lung epithelial cells after different time incubation of cells with nanoparticles loaded with safranin. In addition, nuclei were stained (DAPI), as well as lysosomes (anti-LAMP1 antibody), and all signals were co-localized (merged). Scale bar = 25 μm.
